# Human genetic diversity across South Asian populations: A systematic review and meta-analysis

**DOI:** 10.1097/MD.0000000000050936

**Published:** 2026-10-02

An Expression of Concern is being issued for the article “Human genetic diversity across South Asian populations: A systematic review and meta-analysis,”^[1]^ which appears in Volume 104, Issue 35 of *Medicine*.

Following publication, concerns were raised regarding the methodology and included studies within these systematic reviews, which may affect the reliability of the findings and conclusions presented.

The Journal is investigating these concerns in accordance with Committee on Publication Ethics (COPE) guidance and the journal’s editorial policies.

This Expression of Concern will remain in place while the investigation is ongoing. Upon completion of the investigation, the journal will determine whether further action is required.
